# Do infants use cues of saliva-sharing to infer close relationships? A replication of Thomas *et al*. (2022)

**DOI:** 10.1098/rsos.240229

**Published:** 2025-04-09

**Authors:** Beyza Gokcen Ciftci, Jonathan Frank Kominsky, Gergely Csibra

**Affiliations:** ^1^ Department of Cognitive Science, Central European University, Wien, Austria; ^2^ Maastricht University, Maastricht, The Netherlands; ^3^ School of Psychological Sciences, Birkbeck University of London, London, UK

**Keywords:** social evaluation, infant cognition, saliva-sharing, replication

## Abstract

Thomas and colleagues found in 2022 that the observation of saliva-sharing between individuals serves as an indicator of relationship thickness for children, toddlers and infants. Our study sought to replicate their crucial experiment (2B), which was conducted online and reported that 8.5- to 10-month-old infants anticipated that a crying individual would be more likely to be comforted by a person who had been observed to share saliva with her than another person who simply had played with her. With the exception of changing the testing environment from online to laboratory, and using an eye-tracker supplemented by manual video coding, we closely followed the methodology of the original study. Our replication resulted in partial success: we replicated looking-time preference for the saliva-sharer but did not replicate the tendency to look first to the saliva-sharer upon observing the puppet’s distress. These findings confirm that infants rely on certain behavioural cues for mapping social relationships among third-party individuals.

## 1. Introduction

Understanding social connections is important for children to navigate complex social contexts [1]. During their initial social interactions, infants primarily engage with immediate family members, and the implicit knowledge about parent–child relationships potentially forms the basis for early social understanding [2]. The adaptability of human social cognition requires prioritizing functionally significant social categories [3], where recognizing kinship offers evolutionary advantages by directing affiliative behaviours towards genetically related individuals, increasing inclusive fitness [4,5].

Despite the instability and ambiguity of dynamic small-group interactions, young children demonstrate abilities to infer closeness and affiliation in third-party social relationships, presumably through non-verbal cues such as social signals, affective attitudes and interpersonal behaviours [6]. Studies have shown that, by nine months of age, infants anticipate friendly interactions between individuals who share a common language [7] or food preferences [8]. By 8 to 12 months, infants can expect individuals from the same group to display similar behaviour or choices [9], and toddlers between 15 and 18 months can infer potential affiliations between two adults who comfort the same baby, or two babies comforted by the same adult, indicative of family relationships [2].

Recently, Thomas and colleagues [1] introduced saliva-sharing as a further non-verbal cue that children and infants might use to distinguish ‘thick relationships’ from other positive relationships. They argued that thick relationships encompass a broader concept beyond kinship [10–14], exhibiting fluid associations with genetic relatedness [12,15]. Thick relationships are characterized by strong attachment, mutual obligations and responsiveness, and a sense of unity often expressed as shared bodily substances [16–18]. Saliva-sharing, an interaction observed usually in thick relationships [1,19,20], may help infants and children discriminate between kin and non-kin [21–24] and may contribute to the formation of their early family concepts [1].

To investigate the inferential role of saliva-sharing interactions in third-person relationships regarding thickness, Thomas and colleagues conducted an online study comprising separate experiments involving infants, toddlers and children [1].

In experiment 1, children aged 5 to 7 years were presented with vignettes depicting interactions involving either saliva-sharing (such as licking the same food item) or not (such as sharing partitionable food). The findings suggested that children perceived saliva-sharing interactions as specifically related to nuclear families, while they considered that sharing toys or partitionable food items were equally probable among friends and within families.

In experiments 2 and 3, the study found that inferences about relationship thickness through saliva-sharing might develop at an earlier age, well before the explicit teaching of hygiene rules. The authors familiarized 8.5- to 10-month-old infants and 16.5- to 18.5-month-old toddlers with videos of saliva-sharing and non-saliva-sharing interactions between human actresses and a puppet. During the anticipatory test phase, when the puppet exhibited emotional distress, they measured which actress the participants looked at first, and for a longer duration. Following this phase, infants and toddlers were shown videos of the same actresses engaging in a neutral action (preferential-looking test; ‘peek-a-boo’ in experiment 2) to address an alternative interpretation that children might have a general preference for either of the actresses. This experimental design was based on the assumption that looking behaviour in the anticipatory test phase would indicate infants’ and toddlers’ expectations about who would comfort the puppet. If saliva-sharing interactions serve as a cue for relationship thickness, infants and toddlers might expect the person who has been observed to share saliva with the puppet to comfort it.

In their main experiments (2A for toddlers and 2B for infants), Thomas and colleagues used food-sharing (licking the same orange slice) as a saliva-sharing interaction and ball-passing as a non-saliva-sharing interaction. During the puppet’s distress in the anticipatory test trial, both infants and toddlers tended to look first and longer at the actress who had engaged in saliva-sharing with the puppet, while during the preferential-looking test, they looked equally long at both actresses. To further validate and replicate the initial findings, the authors introduced a series of additional control experiments (e.g. changing the emotional response or changing the nature of the saliva-sharing and non-saliva-sharing interactions).

In experiment 4, a distinct group of parents, whose children were similar in age to those in experiments 2 and 3, participated in a parent survey. The parents were asked, for example, how likely it was that their child used the same straw or cup as the other parent, a grandparent, a babysitter or a friend, and how comfortable they would feel with witnessing such an event. The responses to the survey revealed that parents were comfortable with their children engaging in saliva-sharing interactions only if the child had a thick relationship with the partner. Based on these findings, the authors concluded that saliva-sharing serves as a valid cue for infants and toddlers to infer thick relationships within their social environment, and they can use this particular cue to understand the social dynamics within their social circle.

Assessing how infants and young children map social relations in their environment is a hot topic in developmental psychology, and the findings of Thomas and colleagues may contribute to answering this question. However, these findings have only been replicated by the original authors themselves, and so far no other studies have investigated the role of saliva-sharing interactions in infants’ inference to relationship thickness. In an attempt to validate and extend their results, we conducted a replication of experiment 2B (their main finding with the youngest age group) to examine whether saliva-sharing serves as a reliable cue of relationship thickness for 8.5- to 10-month-old infants.

In our experiment, we used the same video material and the same dependent measures as the original study with some key differences to make the gaze measurement more precise (see table 1 for the summary of the differences and table 2 for commonalities). In case the variance of the dependent variable in this experiment is primarily due to measurement noise, these changes would also make the expected results stronger. Additionally, we integrated the parent survey (used in experiment 4) in our replication as an exploratory measure to determine whether our participant population aligned with that of the original study in terms of their life experiences of bodily intimacy. We did not hypothesize any difference between our own sample and that of the original study.

**Table 1.** Summary of differences between the original and the replication study.

	Original study	Replication study
Testing Environment	Online	Laboratory
Sample size	20 (tested 25)	50 (tested 62)
Gaze Measurement	Manual video coding	Eye-tracker
Sample Population	English speaking	German speaking
Parent Survey	A separate survey study (Experiment 4) was conducted with 129 parents of infants aged 8 to 19 months old, who were not involved in Experiments 2 and 3.	Parents (*n* = 50) whose babies participated in the main experiment (aged between 8.5 and 10 months) completed the survey.
Survey Language	English	English or German
Experimental Design	The same infants participated in Experiment 3B and 2B in the same session (order: first 3B then 2B).	Infants only participated in the experiment corresponding to Experiment 2B of the original study.

The changes in the experimental protocol were made to increase the precision of gaze measurement. We did not expect any difference in the size or direction of the effect resulting from these changes.

**Table 2.** Summary of commonalities between the original and the replication study.

	Original study	Replication study
Stimuli	The original video material and attention-getters from Experiment 2B and the original survey from Experiment 4 were used.	
Procedure	The same order of the phases (Familiarization–Anticipatory Test–Preferential-Looking Test) and the same counterbalancing conditions were implemented with the same amount of time for each video and the phase. For the familiarization phase, the same number of repetitions was applied.	
DVs	The same dependent variables were defined (see §2.8).	
Statistical Analyses	The same statistical tests (descriptives, Bayesian *t*‐test and Bayesian binomial test) were conducted using the same program (JASP).	
Age of Participants	The same age range: 8.5–10 months old.	

## 2. Methods

This article received results-blind in-principle acceptance (IPA) at Royal Society Open Science. Following IPA, the accepted stage 1 version of the manuscript was preregistered on the OSF (https://osf.io/7rcxy/). Data collection started upon receiving no request for modification of the protocol from the reviewers. This preregistration was performed prior to completing data collection and performing analysis. All materials used for performing the study can be found at the OSF repository.

### 2.1. Participants

Fifty full-term infants, aged 8.5 to 10 months, with no reported health or developmental issues, and their parents participated in this study. The sample size was determined before data collection by multiplying the original study’s sample size by 2.5 [25,26]. Infants were recruited through a database to which parents had voluntarily provided contact details for their children’s participation in research.

### 2.2. Ethical approval

Ethical approval for the study was obtained from the Psychological Research Ethics Board of Central European University. Prior to participating in the experiment, caregivers received information about the nature of the study and signed an informed consent form about their child’s participation. At the end of the study, caregivers were provided with a debriefing on the aim of the study and could watch the video recording of their child’s behaviour during the experiment.

### 2.3. Apparatus

The stimuli were presented on a standard 24-inch screen (1920 × 1080 pixels resolution), with participants positioned approximately 60 cm away from the screen. Eye movements were recorded using a Tobii Pro Spectrum eye-tracker, interfaced directly with a Mac Mini computer running the latest version of PyHab [27], a Python-based stimulus presentation software designed for PsychoPy (2023.2.3) [28]. The control of the eye-tracker and the recording of the data were achieved via the ‘psychopy_tobii_infant’ package. Eye-tracking data were recorded with a 60 Hz sampling rate.

### 2.4. Stimuli

The stimuli were obtained from the first author of the original study [1]. This material included video clips featuring two female human actresses and a blue monster puppet acting in the familiarization and test phases, as well as attention-getting stimuli. The full experiment, including stimuli, can be downloaded as a PyHab experiment folder from the OSF repository.

**Figure 1.**
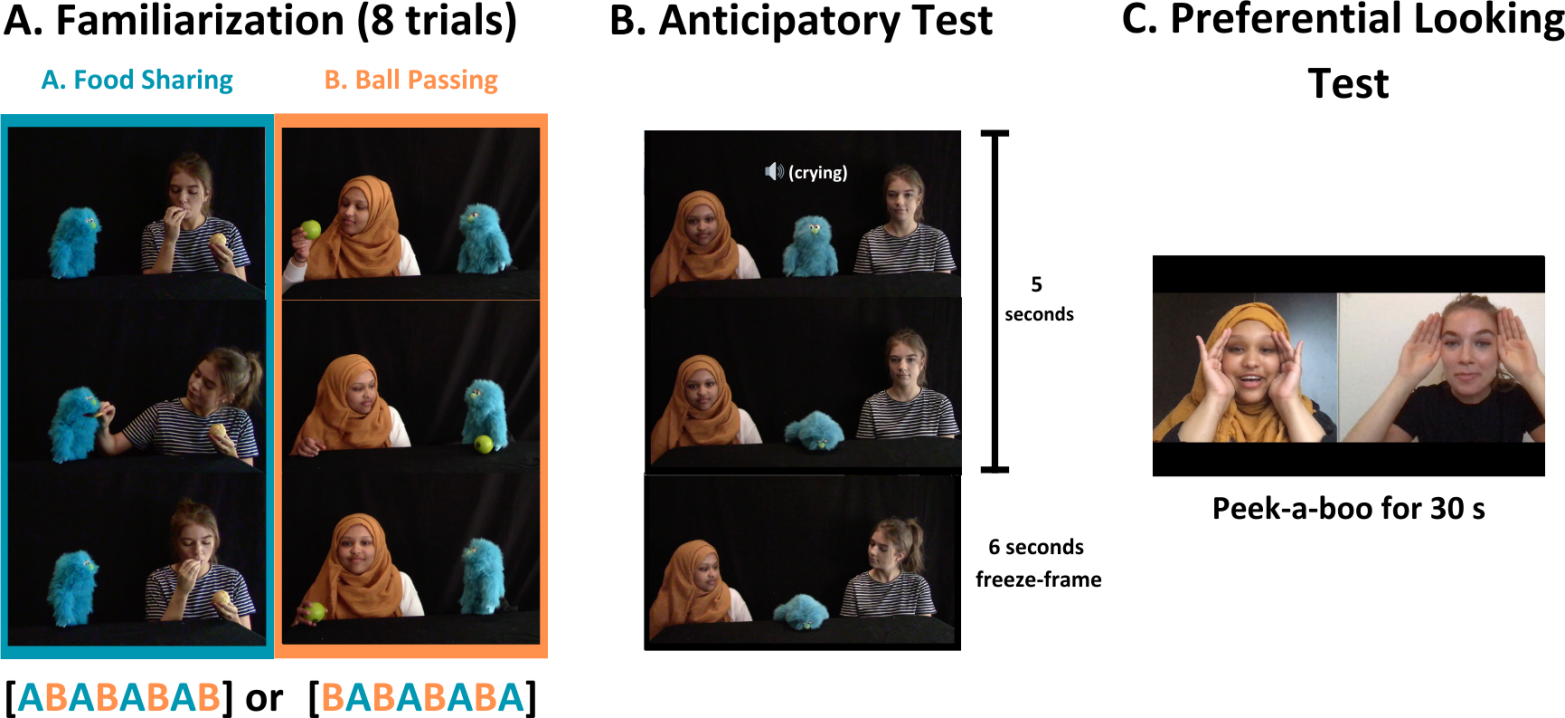
Critical frames of the video stimuli from the three phases of the experiment.

The *familiarization videos* consisted of ‘saliva-sharing’ and ‘ball-passing’ clips, featuring the blue puppet’s interaction with one of the actresses (figure 1A). The background of the videos was black, and the table in front of them was covered with a matching black tablecloth. In the ‘saliva-sharing’ video, the actress took a slice of orange in her hand, placed it in her mouth making a chewing motion, then placed the slice in the puppet’s mouth and the puppet made a chewing motion. Subsequently, the actress placed the slice back into her own mouth. In the ‘ball-passing’ video, the actress took a ball in her hand, looked at it, and then rolled it towards the puppet. Then, the puppet rolled the ball back to the actress, who picked it up again. Both familiarization videos lasted about 12 s (varying between 11.2 and 12.3 s).

The video clip in the *anticipatory test trial* showed the puppet at the centre with the two actresses from the familiarization videos positioned on its right and left (figure 1B). The puppet began crying, which was presented by shaking its body while the sound of a child’s cry was audible, and then placed its head on the table so that its face was covered. Then, both actresses turned simultaneously towards the puppet and the video froze. The clip lasted 5 s and the frozen frame was displayed for a further 6 s.

The stimuli during the *preferential-looking test* showed the faces of the two actresses side by side in separate frames (figure 1C). Throughout the video, the actresses covered and uncovered their eyes with their hands and silently mouthed ‘peek-a-boo’ simultaneously. Between the phases of the study, and within the familiarization phase, different attention-getting stimuli, including a rotating star and a rotating diamond on a checkerboard background, were presented and accompanied by short beeps to attract the participants’ attention (the duration of these stimuli varied between 2 and 5 s).

### 2.5. Procedure

Data collection took place in a laboratory. The procedure involved obtaining informed consent from parents, performing the eye-tracking session in a sound-attenuated room and then asking parents to complete a short survey.

The eye-tracking session consisted of three phases: familiarization, anticipatory test trial and preferential-looking test trial (figure 1). The entire session was video-recorded to allow the evaluation of sessions in which some eye-tracking data were missing. During the experiment, infants were seated on their caregiver’s lap, positioned approximately 60 cm away from a screen in a soundproof room with dimmed lights. Parents were requested to refrain from intervening, maintain silence and wear a pair of opaque glasses throughout the experiment. An experimenter sat behind a curtain and observed infants’ behaviour through a video monitor.

The session started with performing a 5-point calibration of the eye-tracker with pictures of animals sequentially expanding and shrinking in the four corners and the middle of the screen.

The familiarization phase consisted of eight trials, during which the two kinds of video clips (saliva-sharing and ball-passing) were presented in alternation (ABABABAB pattern) with attention-getting stimuli in between. The familiarization phase lasted 2 min. After the familiarization phase, a short attention-getting stimulus was presented, which was then followed by the single anticipatory test trial featuring the crying puppet (5 s video plus 6 s freeze-frame). Finally, during the preferential-looking test phase, the silent peek-a-boo clip was shown repeatedly to the infants for 30 s. The eye-tracking session lasted for about 4 min in total.

### 2.6. Survey

The parent survey [1] was translated into German by native speakers. Caregivers were given the choice to complete the English or German version of the survey. The survey was presented in the same way as in the original study with keeping the original items except for a sentence specific to the pandemic, and demographic questions were removed from the original survey.

We intended to include this survey as an exploratory measure to determine whether our participant population aligned with that of the original study. In the survey, caregivers were asked to identify individuals who have engaged in face-to-face interactions with their infants within the past year. Upon their selection, they were asked to rate the probability of specified interactions occurring between their child and each individual they selected and to assess their comfort levels envisioning their child’s participation in these interactions. Among the interactions, some involved saliva-sharing, while others did not. Caregivers also made similar assessments for themselves and rated the ‘thickness’ of the relationship between the chosen individuals, themselves, and their child.

Parents completed the survey on a tablet in the laboratory after the experiment. MS Word export versions of the survey in both languages can be found at the OSF repository.

### 2.7. Counterbalancing

The side where the two actresses appeared on the screen was the same for every participant and remained constant throughout the whole study.^1^ However, which actress engaged in saliva-sharing with the puppet and which one interacted through ball-passing was counterbalanced: half of the infants watched actress A sharing food with the puppet and actress B playing ball, while the other half watched actress B sharing food and actress A engaging in ball-passing. In this way, the positioning (right or left) of the saliva-sharing actress was counterbalanced across participants during the familiarization, anticipatory test and preferential-looking test trials. Additionally, which type of interaction infants observed first (either saliva-sharing or ball-passing) during the familiarization phase was also counterbalanced.

### 2.8. Dependent measures

**Figure 2.**
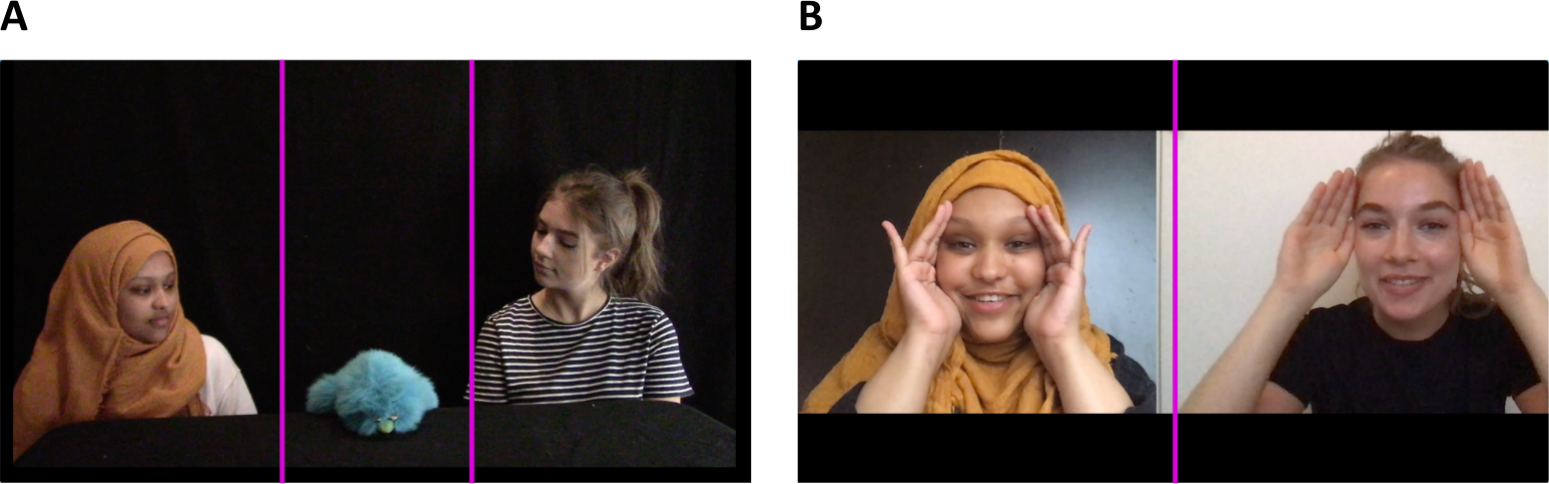
Areas of interest for measuring gaze in the anticipatory test phase (A) and in the preferential-looking phase (B).

The main dependent measures evaluated infants’ gaze during the anticipatory test trial. To quantify looking behaviour, two areas of interest (AOIs) were defined corresponding to the two sides of the screen showing the two actresses (the saliva-sharer and the ball-passer). These two AOIs occupied the left and right 3/8 s of the screen in full height while leaving the middle 1/4, where the puppet was located out (see figure 2A). Regarding pixel coordinates on the 1920 × 1080 screen with the centre considered as (0,0), any X (the horizontal coordinate of the gaze position) greater than 240 corresponds to the right AOI, while any X lower than −240 corresponds to the left AOI. With these AOIs, we defined two measures of anticipation (first look and duration). As in the original study [1], the primary dependent measure was the first gaze infants made into either AOI starting from the freeze-frame. If an infant fixated on either AOI at the time of the freeze-frame and continued to keep her gaze there for more than 0.4 s, we recorded this, in line with the original study, as their first look. This was a binary dependent measure.

The secondary dependent measure to test the main prediction was the proportion of infants’ looking time at the saliva-sharer following the puppet’s expression of distress during the anticipatory test trial. To calculate this measure, we obtained the number of frames the infants’ gaze spent in either AOI during the 6 s starting the freeze-frame of the video and divided this value for the saliva-sharer’s AOIs by the sum of both values.

Note also that we measured eye movements during the entire anticipatory test trial, and not just from the freeze-frame. This approach allowed us to perform exploratory measures of gaze in order to assess the possibility that infants would preferentially look at one of the actresses before or during their head turn towards the puppet.

In addition to measures of anticipation, we also quantified infants’ preference for the actresses during the preferential-looking phase. This measure was calculated similarly to the primary dependent measure, except that the two AOIs comprised the two halves of the screen (figure 2B), and the measurements lasted the entire duration of the video (30 s).

### 2.9. Exclusion criteria

Participants were excluded based on predetermined criteria. Infants were excluded from the analysis (a) if they became fussy during the experiment and were not able to complete it, (b) if caregivers intervened during the procedure, (c) if a technical failure occurred during stimulus display or video recording, (d) if the participant looked at each type of familiarization video for less than 15 cumulative seconds across the 4 presentations, (e) if they looked away more than 100 ms consecutively in the test trials before the freeze-frame, or (f) if they did not look away from the puppet during the 6-s freeze-frame in the anticipatory test trial.

In addition, exclusion criteria regarding eye-tracking failures included (g) less than 50% proportion of usable data during the anticipatory test trial after the freeze-frame. A valid sample for the eye-tracker data was defined as a valid measure from at least one eye with an X-coordinate between −1000 and 1000 and a Y-coordinate between −600 and 600. Samples outside those values were excluded as these fall outside the boundaries of the screen with a small margin of error for imprecise calibration.

Excluded participants were replaced to obtain the planned number of participants with usable data. We report below the number of excluded participants for each of the above criteria.

### 2.10. Confirmatory statistical analyses

Based on the original study, we hypothesized that infants would look first and longer to the saliva-sharer compared with the ball-passer during the freeze-frame in the anticipatory test trial. Additionally, we hypothesized that the looking behaviour observed in the preferential-looking test would not reveal a preference for either of the actresses. We reproduced the original study’s analyses using R and JASP, reporting the results in the same format as the default Bayes factors.

For the first hypothesis (infants look first and longer to the saliva-sharer than to the ball-passer during the anticipatory test trial), we used the same dependent variables as the original study: first look and proportion of looking duration. To check whether more infants looked at the saliva-sharer first than chance, we used a two-sided Bayesian binomial test. To analyse whether the proportion of time infants spent looking at the saliva-sharer during the freeze-frame was different from 0.5 (the null hypothesis), we performed a Bayesian two-sided one-sample *t*‐test. We considered these two tests as separate measures, without giving preference to one over the other, as our aim was to identify which patterns of results in our experiment aligned with those of the original study.

For the second hypothesis, to explore whether infants had specific preferences for either actress, the proportion of time on the saliva-sharer was compared to chance (0.5) using a Bayesian two-sided one-sample *t*‐test.

### 2.11. Predefined interpretation of the findings

The success of the replication attempt was evaluated individually for the two dependent variables of the anticipatory test trial, along with the findings of the preferential-looking test.

For the first-look analyses to be considered successfully replicated, we expected to yield strong evidence in favour of the alternative hypothesis (BF₁₀ ≥ 10). In contrast, the results were planned to be judged as a replication failure if they provided substantial evidence in favour of the null hypothesis (BF₀₁ ≥ 3). The same criteria were applied for the analysis of proportion of looking times with the proviso that the judgment of successful replication here was also dependent on finding substantial evidence (BF₀₁ ≥ 3) in favour of the null hypothesis in the preferential-looking test. These thresholds were established based on the strength of the evidence in the original study (BF₁₀ = 10.306 for the first looks and BF₁₀ = 76.861 for looking proportion in favour of the alternative hypothesis; BF₀₁ = 3.918 in favour of the null hypothesis in the preferential-looking test) [1]. Any Bayes factor values falling between these thresholds were considered inconclusive for determining replication success or failure.

### 2.12. Exploratory statistical analyses

To further evaluate not just whether we find an effect but whether our results closely replicate the original results, we included the replication Bayes factor approach in our analyses. The default Bayes factor analyses yield the probability of our data under the alternative hypothesis over the null hypothesis when there is no *a priori* expected effect size in the replication attempt. The replication Bayes factor analyses indicate whether the support for the alternative hypothesis is similar to the one found in the original study or not. In this approach, the null hypothesis (skeptic’s hypothesis) posits that the effect is absent, while the alternative hypothesis (proponent’s replication hypothesis) suggests that the effect is consistent with the original study’s effect [29]. Note that this cannot be used for comparisons that did not show a significant effect in the original study (e.g. the preferential-looking test trial), because the proponent’s and skeptic’s hypotheses are the same (i.e. no effect).

We computed Bayes factors in the same way as above except for updating the priors based on the posterior distribution of the original study. For the Bayesian two-sided binomial test, we defined the priors by using the first-look distribution of experiment 2B of the original study. For the Bayesian two-sided one-sample *t*-test (for proportions of looking time in the anticipatory test trial), we used the median and confidence intervals of the posterior distribution of looking proportions of the same experiment. With these informed priors, we computed replication Bayes factors [29].

Moreover, as part of our exploratory analysis, we descriptively compared the original survey results with the current survey results to check for potential ecological differences between the two populations.

## 3. Results

Based on the predefined criteria, we excluded 12 participants due to fussiness (*n* = 6), eye-tracker problems (*n* = 2), caregiver intervention (*n* = 1) or missing 100 consecutive milliseconds before the freeze-frame during the anticipatory test trial (*n* = 3). The age of the 50 infants (24 females and 26 males as indicated by their parents) who were kept in the analyses ranged from 259 to 306 days (mean age was 279.32 days; s.d. = 12.95 days).

All the anonymized data and the analysis scripts are available at the OSF repository.

### 3.1. Main analyses

**Figure 3.**
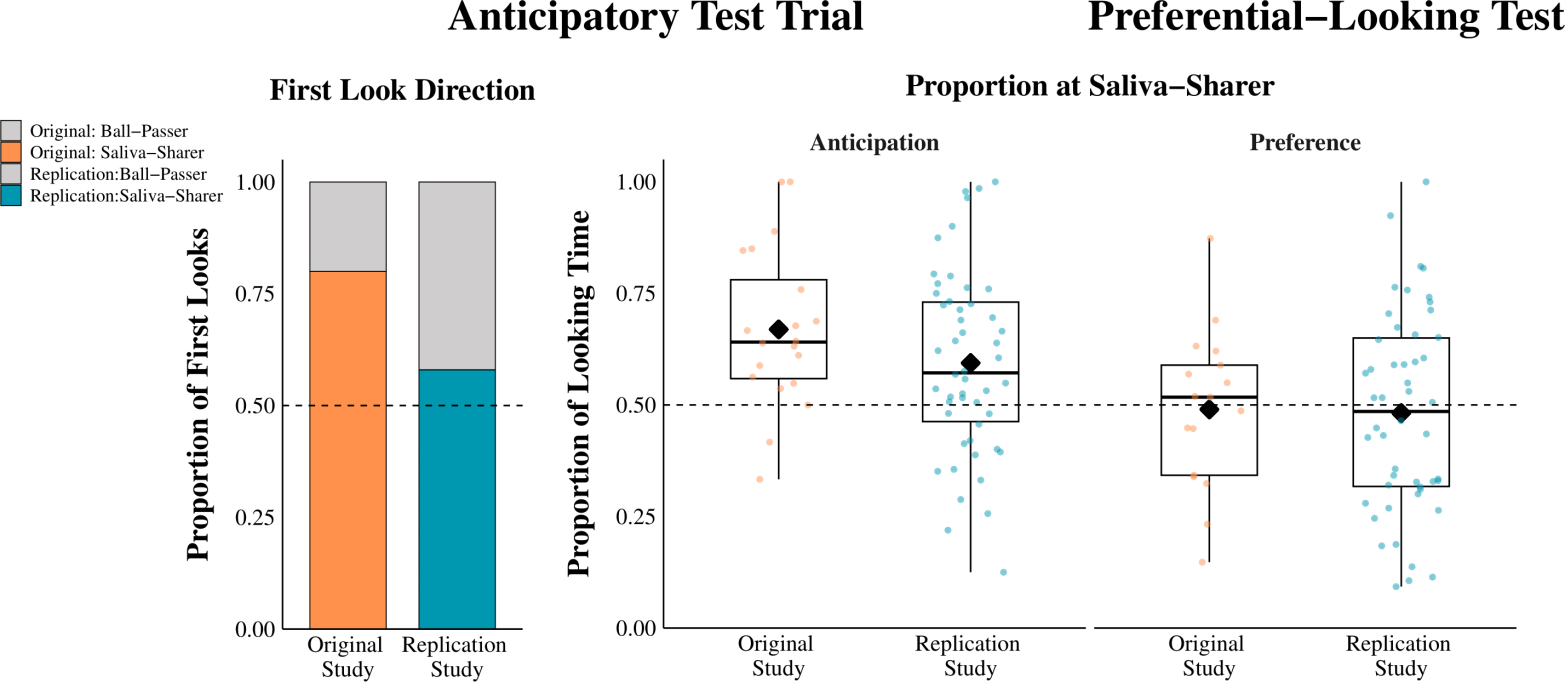
Comparisons of the main findings between Thomas *et al*. [1] and the present study. The bar graph on the left represents the proportion of infants who looked first at the target (the saliva-sharer) during the anticipatory test trial. The box plots on the right display the proportions of looking time at saliva-sharer during the anticipatory test trial and preferential-looking test. Black diamonds indicate the means, bars indicate the medians, and the dots represent individual proportion of looking time at the saliva-sharer. The data from the original study were extracted from its OSF repository (https://osf.io/a8htx/files/osfstorage).

Figure 3 summarizes our main findings and contrasts them to those of the original study.

Of the 50 infants, 29 (58%) looked first at the saliva-sharing actress from the moment the video froze in the anticipatory test trial. A Bayesian binomial test revealed substantial evidence for the null hypothesis (BF₀₁ = 3.05), suggesting that the primary dependent measure showed no significant deviation from the chance level (50%). Thus, we did not replicate the original finding, where a large majority of infants (80%) looked first at the saliva-sharing actress upon having been confronted with the emotional distress of the puppet.

Concerning the secondary dependent measure, infants spent more time looking at the saliva-sharer than at the ball-passer (mean proportion at saliva-sharer = 0.59; s.d. = 0.21), which, according to a Bayesian one-sample *t*‐test (BF₁₀ = 13.62), provided strong evidence in support of the alternative hypothesis. Thus, our secondary dependent measure did replicate the finding reported by Thomas *et al*. [1].

In the preferential-looking control trial, infants spent on average 0.48 (s.d. = 0.22) proportion of time looking at the saliva-sharer. According to a Bayesian one-sample *t*‐test, this result provided substantial evidence in favour of the null hypothesis, BF₀₁ = 5.54, suggesting no preference for either actress. Thus, our experiment replicated the finding of the original study: the preference for the saliva-sharer in the main test trial cannot be explained by a general preference for the saliva-sharing actress.

### 3.2. Exploratory analyses

As an additional exploratory measure, we looked at the proportion of times infants shifted their gaze towards the saliva-sharer (compared to gaze shifts towards either actress), either from the puppet or from the other actress. The proportions of switches were calculated similarly to the proportion of looking time. A non-directional Bayesian one-sample *t*‐test indicated that infants looked more frequently towards the saliva-sharer than to the ball-passer (mean proportion of switch to saliva-sharer = 0.56, s.d. = 0.15, BF₁₀ = 4.85).

We also examined infants’ first look direction from the beginning of the anticipatory test trial (as opposed to the freeze-frame): 31 of 50 infants looked first to the saliva sharer (BF₀₁ = 1.38), which is inconclusive evidence for either hypothesis. When we analysed infants’ looking proportions over the entire anticipatory test trial (rather than just from the onset of the freeze-frame), the results remained consistent with the main analysis and showed support for the alternative hypothesis (*M* = 0.59; s.d. = 0.19; BF₁₀ = 27.49).

To investigate whether the effects in the anticipatory test trial were due to differential attention to the two actresses during familiarization, we measured and compared the proportion of time the infants looked off-screen while watching the two videos. A Bayesian non-directional paired sample *t*‐test revealed no significant difference in the proportion of off-screen looks between saliva-sharing videos (mean proportion = 0.07, s.d. = 0.08) and ball-passing videos (mean proportion = 0.05, s.d. = 0.06), BF₁₀ = 2.30.

To compare the effect in the two studies, we calculated the effect size for the two main dependent variables. For the first-look directions, we used Cohen’s *g* as an effect size measure (observed proportion–null proportion) [30]. The effect size in the replication study was *g* = 0.08, while in the original study, it was *g* = 0.3. For the proportion of looking times at the anticipatory test trial, we used Cohen’s *d* as an effect size measure. The effect size of the original study (*d* = 0.95, s.e. = 0.27, 95% CI [0.41, 1.47]) was twice as big as the replication study (*d* = 0.46, s.e. = 0.15, 95% CI [0.16, 0.74]). However, the 95% confidence intervals overlap, indicating that the differences between the studies are not statistically significant. Despite the large differences in the magnitude of effect size between the two studies, both showed a statistically significant preference in the same direction.

#### 3.2.1. Replication Bayes factors

The replication Bayes factor analysis for the first-look data yielded support for the skeptic’s hypothesis (BF₀₁ = 4.00). This indicates that our results did not find the effect on first looks that was reported in the original study.

For the proportion of looking times during the anticipatory test trial, the replication Bayes factor analysis indicated support for the proponent’s replication hypothesis (BF₁₀ = 22.90). This means that the effect found in the replication study was similar to the original study.

#### 3.2.2. Manual video codings

One of the differences between the original and the replication study was that we measured infants’ gaze direction by an eye-tracker, while in the original study, the dependent variables were quantified by manual coding of gaze direction from the recording of the online session. To compare the two methods, we manually coded the recorded videos as well. The results of the proportion of the first looks remained consistent with the main analysis (30 of 50 infants looked first at the saliva-sharer, BF₀₁ = 2.14). For the proportion of looking time measure, we found stronger evidence for the alternative hypothesis (*M* = 0.59; s.d. = 0.18; BF₁₀ = 25.74), and for the preferential-looking test, the support was quite similar to the eye-tracker findings (BF₀₁ = 5.83).

### 3.3. Parent survey analyses

Of the parents of the 50 infants who participated in, and completed, the study, we collected survey responses from 48 parents (1 parent did not participate in the survey and 1 parent only answered the first question due to language problems).

Bayesian correlation analyses provided no support for any correlation between infants’ proportion of looking time to the saliva-sharer and the parent survey responses. Infants’ proportion of looking times was not related to their parents’ comfort level with their participation in saliva-sharing interactions or other interactions, infants’ likelihood of participation in these interactions, or their likelihood of observing their parents’ participation in these interactions both in general and when categorized by kinship and relationship closeness.

However, our survey findings showed similar patterns to the original study in terms of parents’ comfort levels with their children’s participation in saliva-sharing and non-saliva-sharing interactions, parents’ ratings of their children’s likelihood of participating in these interactions and children’s likelihood of observing their parents participating in these interactions. There were no differences between the original and replication studies regarding the direction of difference between saliva-sharing and non-saliva-sharing interactions. However, there were minor differences in terms of the magnitude of means between the two studies (see OSF repository for more detailed information about the survey results).

## 4. Discussion

In our replication attempt, we partially replicated the findings of Thomas *et al*. [1]: the proportion of looking time to the saliva-sharer confirmed the hypothesis of the original study (and the control measure indicated that this was not due to an unconstrained preference for her), but the tendency to look first to the saliva-sharer upon observing the puppet’s distress failed to show a similar effect. In the Thomas and colleagues’ study, 16 out of 20 infants (80%) looked at first to the saliva-sharer, while in our study with 50 infants, only 29 of them looked at first to the saliva-sharer (58%). While this outcome was not different from chance level responding, we found clear support for the original study in the looking time proportions. Like in the original study (*M* = 0.67), in our sample, infants looked more to the saliva-sharer in the anticipatory test trial (*M* = 0.59) and they did not display any preference for the saliva-sharing actress in the preferential-looking test (original study: *M* = 0.49; present study: *M* = 0.48). The replication Bayes factor analyses and manual video coding analyses were consistent with these findings as well.

In addition, we did not find a difference in infants’ attention between saliva-sharing and ball-passing interactions during the familiarization phase. In this way, we have eliminated an alternative explanation that infants may have looked longer at the saliva-sharing actress in the anticipatory test trial because they attended to her more in the familiarization trials.

The results of the parent survey did not reveal any differences from the original study regarding infants’ daily life experiences of bodily intimacy, suggesting that our Austrian sample was similar to Thomas *et al*.’s [1] US sample in this regard. Since we collected survey data from the caregivers of the babies participating in the study, we could directly compare the infants’ gaze behaviours with the survey data. We did not find any relationship between the infants’ gaze behaviour and the parents’ survey responses.

In our replication attempt, we used an eye-tracker to make the gaze measurement more precise. However, when we compared the eye-tracking data with the manually coded gaze data, we found similar results. This has two implications. First, this suggests that, in the original study, the variance of the dependent variable was unlikely due to measurement noise. Second, it indicates that for simple dependent measures, like the one used in this experiment, manual coding is a sufficiently reliable method of measurement.

One may wonder why the two dependent measures (first-look directions and looking time proportions) that targeted the main hypothesis of the study diverged in confirming the original findings. This discrepancy is consistent with the argument that a continuous measure based on multiple samples within a single trial is more likely to be a reliable measure and produces a higher effect size than the one that depends on a single decision [31]. In the anticipatory test phase of our study, both the first look and the looking time proportion measures indicated a 58–59% preference for the actress who shared saliva. However, only the continuous measure withstood statistical scrutiny. These findings support the recommendation of focusing on the proportion of looking times, instead of the first-look outcomes, as a primary dependent measure of anticipation in infancy.

While our replication study has provided partial support for the reliability of the findings of Thomas *et al*. [1], it is important to mention that we did not address the question of how to interpret these results. In particular, we think that saliva-sharing is not the only possible cue that might have prompted infants to expect one of the actresses to intervene when the puppet started crying. In the familiarization videos, the ‘saliva-sharer’ actress and puppet chewed the same orange slice sequentially, but the presence of saliva was not visually accessible in the videos. It is, thus, not clear which element of this sequence was crucial for infants to infer a thick relationship. For example, the study did not test what would happen if the actress placed the orange slice in the puppet’s mouth without first chewing it herself (feeding without saliva-sharing) or if the actress chewed the orange slice, placed it on the table, and then the puppet took the slice by itself and chewed it (saliva-sharing without feeding). After all, feeding (even without saliva-sharing) might also be indicative of a thick relationship between the individual who provides the food and the one who receives it. Feeding is easier to recognize than saliva-sharing and might also correlate strongly with the strength of social relationships. We acknowledge that Experiment 3 of Thomas *et al*. [1] tested the saliva-sharing hypothesis without visible food items, but since mouth-to-mouth feeding (premastication), which transfers only a small amount of pureed food between individuals, is widespread among species and human societies, moving fingers from mouth to mouth is an action that is compatible with the purpose of feeding.

Still, our findings regarding the partial replicability of the original study indicate that infants at this age can represent and map social relationships (whether saliva-sharing cues are the critical feature or not). Importantly, our findings demonstrate that at least one of the dependent measures of the original study is robust, which makes it a good paradigm for future work in this area. Researchers can have confidence that if a modified display yields different results, it is due to the modification and not to the paradigm itself being unreliable.

Together, the present research not only provides further support for our understanding of how infants and young children map social relationships in their environment but also offers valuable methodological insights for studies with infant participants. It emphasizes the importance of defining the dependent variables, selecting the measurement methods and employing reliable paradigms. Future studies that better control the saliva component of the main hypothesis of the Thomas *et al*. [1] study could further clarify the specific role of saliva-sharing in infants’ inferences to relationship thickness.

## Data Availability

All the data and the analysis scripts are available at the OSF repository [32].

## Footnotes

^1^
The stimulus examples available at the OSF site for the original study [1] include videos in which the two actresses swap positions for the preferential-looking test. We were informed by the authors that this was a mistake and the positions of the actresses never changed throughout the study.
